# Simultaneous delivery of olaparib and carboplatin in PEGylated liposomes imparts this drug combination hypersensitivity and selectivity for breast tumor cells

**DOI:** 10.18632/oncotarget.25466

**Published:** 2018-06-19

**Authors:** Vojtech Novohradsky, Juraj Zajac, Oldrich Vrana, Jana Kasparkova, Viktor Brabec

**Affiliations:** ^1^ Institute of Biophysics, Czech Academy of Sciences, Kralovopolska 135, CZ-61265 Brno, Czech Republic

**Keywords:** olaparib, carboplatin, combination, liposomes, antitumor activity

## Abstract

Combination regiments involving platinum anticancer drugs and agents with unrelated mechanisms of action are a subject of widespread interest.

Here, we show that synergistic toxic action in cancer cells of combinations of antitumor platinum drug carboplatin and effective PARP inhibitor olaparib is considerably improved if these combined drugs are encapsulated into liposomes. Notably, the formation of such nano-formulations, called OLICARB, leads to a marked enhancement of activity in human cancer cell lines (including those resistant to conventional platinum antitumor drugs) and selectivity towards tumor cells. We used immunofluorescence analysis of γH2AX expression and examined DNA damage in cancerous cells treated with the investigated compounds. We find that the synergistic toxic effects in cancer cells of both drugs used in combination, nonencapsulated or embedded in the OLICARB nanoparticles, positively correlates with DNA damage. These results also suggest that the enhancement of the toxic effects of carboplatin by olaparib in cancer cells is a consequence of an accumulation of cytotoxic lesions in DNA due to the inhibition of repair of platinated DNA augmented by the synergistic action of olaparib as an effective PARP inhibitor. Our findings also reveal that the combination of olaparib with carboplatin encapsulated in the OLICARB nanoparticles is particularly effective to inhibit the growth of 3D mammospheres. Collectively, the data provide convincing evidence that the encapsulation of carboplatin and olaparib into liposomal constructs to form the OLICARB nanoparticles may represent the viable approach for the treatment of tumors with the aim to eliminate the possible effects of acquired resistance.

## INTRODUCTION

Combination chemotherapy for the treatment of cancer was introduced approximately 50 years ago [1, 2]. The rationale for combination chemotherapy is to use drugs that work by different mechanisms, thereby decreasing the likelihood that resistant cancer cells will develop. There are many combinations of chemotherapy drugs that are used for different types of cancer. A number of metallodrug–organic drug combinations have already been reported [3]. Many metallodrugs coordinatively bind to DNA forming adducts, while organic drugs tend to interact with signaling pathways, inhibiting protein synthesis and potentially affect DNA repair mechanisms [4]. Combining metal- and organic-based drugs to increase therapeutic efficacy over single drug treatments, that is to produce a synergistic effect, has the potential to aid in overcoming resistance by targeting different mechanisms of action [5]. This therapeutic approach has significant implications for metallodrugs. For instance, the strategy of using co-treatment with clinically used platinum cytostatics with inhibitors of repair of damaged DNA has made it feasible to overcome platinum-based drug resistance so that these inhibitors sensitized some tumor cells to platinum drugs [6, 7]. The results of related preclinical testing [8–11] demonstrated increased antitumor activity of platinum drugs combined with inhibition of the enzyme poly ADP ribose polymerase (PARP) pointing to a role of PARP proteins in the repair of DNA damaged by platinum antitumor drugs.

PARP comprises a family of proteins which regulate a number of cellular processes, among them also DNA repair [12]. Among mammalian DNA repair pathways, PARP proteins, in particular PARP-1, have been implicated in base excision repair, homologous recombination, nonhomologous end-joining pathways and also in the most versatile nucleotide excision repair (NER) pathway [13–15]. Interestingly, NER is the main pathway used by tumor cells to remove major DNA lesions induced in DNA by cisplatin and its clinically used derivatives [16, 17].

The PARP inhibitors block the activity of this enzyme, thus preventing the repair of DNA damage and its persistence and ultimately causing cell death. Besides, it has been established [18] that PARP inhibitors have an additional mode of action: localizing PARP proteins at sites of DNA damage, which has relevance to their antitumor activity. The trapped PARP protein–DNA complexes are highly toxic to cells because they block DNA replication. Interestingly, PARP-1, most studied PARP, has been identified as a platinum-DNA damage response protein [19]; the amount of protein that binds to the most frequent DNA lesion (1,2-GG intrastrand cross-link) of cisplatin and its derivatives was found greater than the amount that binds to other types of cisplatin-DNA adducts [20].

One of the effective PARP inhibitors is olaparib [21], which is the best-studied PARP inhibitor to date. Preclinical data suggest that olaparib might potentiate the efficacy of DNA-damaging chemotherapies, including clinically used platinum-containing drugs [9, 22–25]. It is so because the PARP inhibitors block the activity of this enzyme, thus preventing the repair of DNA damage caused by carboplatin and its persistence. It is a well-known fact that the lesions formed on DNA by platinum antitumor drugs, such as cisplatin and carboplatin, are responsible for antitumor effects of these drugs, but simultaneously these DNA lesions can also be effectively removed by DNA repair machinery. Thus, the uninhibited repair of platinum-DNA lesions can produce cells that resist to platinum anticancer drugs [16, 26], or, in other words, inhibition of repair of lesions formed on DNA by platinum antitumor drugs can considerably increase the sensitivity of tumor cells to these metallodrugs. Recent trials assessing olaparib in combination with platinum drugs in patients with advanced ovarian, breast and other solid tumors have shown encouraging efficacy as well [27–29].

Combination chemotherapies, despite many advantages, suffer from drawbacks. Examples are optimization of concentrations of the combined drugs, duration and timing of the treatment. These problems may be a consequence of the different pharmacokinetics, cellular accumulation and intracellular distribution of the drugs used in combination. Rationalized approaches how to overcome these shortcomings are based on using nanoparticles as carriers of the combined drugs [30]. Conceivably, the efficacy of a platinum drug in combination with olaparib might be augmented if both drugs are co-encapsulated in nanoparticles. To investigate this hypothesis at the cellular level, we prepared PEGylated liposomal nanoparticles with encapsulated olaparib and carboplatin at their defined molar ratios. We treated with these nanoparticles human tumor cells with acquired or inherited resistance to cisplatin and noncancerous cells cultured in monolayer or as 3D cell cultures (spheroids). We found that the synergistic antitumor effects of the two agents and selectivity for tumor cells were markedly enhanced when co-encapsulated into liposomes at the defined molecular ratio. Taken together, our results point to a combined chemotherapy connected with nanotechnology which shows great promise in the treatment of cancer overcoming drug resistance.

## RESULTS AND DISCUSSION

### Preparation and characterization of nanoparticles

**Figure 1:**
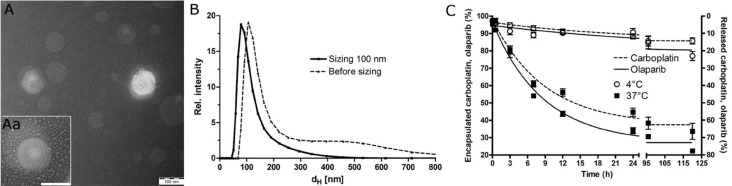
Characterization of the investigated nanoparticles (**A**) A typical TEM image of OLICARB_1:1_. Scale bar represents 100 nm. Aa. Detailed TEM image of a single liposomal nanoparticle OLICARB_1:1_. (**B**) Hydrodynamic diameter (*d*H) measured by DLS; suspension of OLICARB nanoparticles after sizing (full line) and before sizing (dashed line). (**C**) The release profile of carboplatin (dashed line) and olaparib (full line) from liposomes tested in the DMEM medium at 4° C and 37° C (pH 6.8).

We prepared PEGylated liposomes to improve not only the stability, circulation time and bioavailability but also the ‘passive’ targeting to tumors, through the enhanced permeation retention effect, which improves the therapeutic effects and reduces the toxicity of encapsulated drugs [31]. The liposomal nanoparticles (OLICARB) containing encapsulated combinations of olaparib and carboplatin at the ratio of their molar concentrations of 1:1 and 2:1 (OLICARB_1:1_ and OLICARB_2:1_, respectively) were characterized by inductively coupled plasma mass spectrometry (ICP-MS) or flameless atomic absorption spectrometry (FAAS) for platinum content, UV absorption spectrophotometry for olaparib content; the phosphate analysis for the phospholipid content was performed by ICP-MS. This analysis yielded atomic ratios of Pt/P of 1:19.7 and 1:15.9 for OLICARB_1:1_ and OLICARB_2:1_, respectively. Size analysis by transmission electron microscope (TEM) (Figure 1A) showed a typical ring-shaped morphology with high density in the center of phospholiposome [32]. The unilamellar vesicles were also confirmed by the TEM. The mean hydrodynamic diameter (*d*_H_) of OLICARB nanoparticles before sizing was 182±8 nm. The OLICARB nanocapsules with a narrower size distribution were obtained as described in the experimental part by extrusion through polycarbonate filters with 100 nm pore size. Extrusion through small pores breaks up multilamellar vesicles into smaller vesicles that are comparable to the pore size [33]. Size analysis by dynamic light scattering (DLS) revealed that the main fractions of these filtered nanocapsules contained particles with hydrodynamic diameters of (107 ± 6) nm (mean ± standard deviation (SD)) (Figure 1B); zeta potential (ξ_p_) value was determined to be −38 mV suggesting that the OLICARB nanoparticles were stable in suspension and well suited to take advantage of the enhanced permeability and retention effect (EPR) [34, 35].

### *In vitro* controlled release of platinum and olaparib from encapsulated nanoparticles

The controlled release kinetics of olaparib and platinum from carboplatin from OLICARB nanoparticles in the cell culture medium (Dulbecco’s modified eagle’s medium, DMEM, pH 6.8 and 7.4) at 37° C and 4° C were examined as well (shown for OLICARB_1:1_ in Figure 1C and Supplementary Figure 1). The OLICARB nanocapsules were stable without the detectable release of platinum or olaparib at 4° C for at least 24 h. Under the physiological temperature (37° C), a considerable release of the encapsulated compounds was confirmed from both OLICARB_1:1_ and OLICARB_2:1_; for instance, the total amount of the released platinum from carboplatin from OLICARB_1:1_ after 24 h of incubation at pH 6.8 was ∼57%, and that of olaparib was ∼63%; the total amount of the released platinum from carboplatin from OLICARB_1:1_ after 24 h of incubation at pH 7.4 was somewhat lower, ∼43%, and that of olaparib was ∼55%. These results demonstrated a sustainable and continual release of both encapsulated compounds from the OLICARB nanoparticles, a prerequisite for biological (antitumor) activity [36].

### Cytotoxicity

The cytotoxic activity was first determined for free carboplatin and olaparib and their mixture (molar ratio of olaparib:carboplatin was in the range 1:3–3:1) against the panel of four human cancer cell lines and one non-malignant cell line (Table 1). These experiments were also performed to determine the optimal ratio of olaparib:carboplatin for their encapsulation into PEGylated liposomes. The cytotoxicity was evaluated against a group of human cancer cell lines, including human ovarian carcinoma cell lines A2780 (cisplatin sensitive) and A2780cisR (with acquired resistance to cisplatin), the breast tumor cell lines MCF-7 and MDA-MB-231 (highly invasive, triple negative). These cancer cell lines were chosen as the representatives of typical human malignancies for which carboplatin and/or olaparib has been approved for the clinical use and are also commonly used to test cytotoxic activity of cisplatin, its derivatives, and other antitumor metallodrugs.

**Table 1:** Cytotoxicity of olaparib and carboplatin used to treat cancer and noncancerous cells as single drugs or in combination (as the mixtures of these drugs)^a^

IC_50_^*b*^ (μM)	Free drugs		Combination of free drugs (Olaparib:Carboplatin)				
	Olaparib	Carboplatin	1:3	1:2	1:1	2:1	3:1
A2780	5.7 ± 0.7	21 ± 2	15 ± 2	5.1 ± 0.5	2.9 ± 0.1	2.1 ± 0.2	1.7 ± 0.1
A2780cis	30 ± 3	83 ± 5	47 ± 4	38 ± 3	22 ± 4	7 ± 1	9 ± 2
MCF-7	54 ± 4	73 ± 5	72 ± 6	53 ± 5	23 ± 3	17 ± 2	16 ± 1
MDA-MB-231	37 ± 3	151 ± 12	83 ± 7	83 ± 5	32 ± 6	28 ± 3	20 ± 2
MRC5 pd30	152 ± 14	142 ± 4	29 ± 1	18.4 ± 0.3	68 ± 2	42 ± 2	42 ± 5

^a^ The cells were incubated with the drugs or their combinations for 72 h.

^b^The results are expressed as mean values ± SD of three independent experiments, each made in triplicate. The IC_50_ values for drug combinations are related to the concentration of carboplatin.

As shown in Table 1, olaparib was more cytotoxic than carboplatin in all cancer cell lines. Both olaparib and carboplatin were less toxic in cisplatin-resistant cells A2780cisR (exhibiting acquired resistance) and breast cancer cells (inherently resistant) in comparison with their toxicity in A2780 cells sensitive to cisplatin.

The treatment with carboplatin combined with olaparib led to an increase in the toxicity of carboplatin not only in cisplatin sensitive A2780 cells but also in the cancer cells resistant to cisplatin (4-12-fold if the molar ratio of olaparib:carboplatin was 2:1 or 3:1). Fundamentally different effects were observed if noncancerous cells MRC5 pd30 were treated with the mixtures of the drugs. The low ratios of olaparib:carboplatin (1:3 or 1:2, i.e., when concentrations of olaparib were lower than that of carboplatin) resulted in the increase of the toxicity of carboplatin (5-8-fold). In contrast, a markedly lower increase of toxicity was noticed if MRC5 pd30 cells were treated with higher ratios of olaparib:carboplatin (3:1 or 2:1, i.e., when the concentrations of olaparib were higher than that of carboplatin).

The results shown in Table 1 indicate that the highest enhancement of toxicity of carboplatin in cancer cell lines, when combined with olaparib, was achieved if the ratio of their molar concentrations (olaparib:carboplatin) was higher than 1. Therefore, next, we prepared the mixtures of these drugs encapsulated in the PEGylated liposomes so that the ratio of their molar concentrations (olaparib:carboplatin) in nanocapsules was 1:1 or 2:1 (OLICARB_1:1_ or OLICARB_2:1_, respectively) and investigated the efficiency of the encapsulation to promote cytotoxicity. The results shown in Table 2 demonstrate that toxicity of the combinations of olaparib and carboplatin encapsulated in the PEGylated liposomes (OLICARB_1:1_ and OLICARB_2:1_) in cancer cell lines was markedly increased (3-13-fold) in comparison with free (nonencapsulated) mixtures of the drugs. The values of IC_50_ (IC_50_ = concentration of the agent inhibiting cell growth by 50%) in A2780 cells were even in the submicromolar range, and notably, the most positive effects of encapsulation on cytotoxicity were observed for cisplatin-resistant cancer cells A2780cisR and MDA-MB-231.

**Table 2:** Cytotoxicity of the OLICARB nanoparticles (mixtures of olaparib and carboplatin encapsulated in PEGylated liposomes) or olaparib and carboplatin encapsulated as single agents in cancer and noncancerous cells^a^

IC_50_ (μM)^*b*^	OLICARB_1:1_^*c*^	OLICARB_2:1_^*c*^	Olaparib_NANO_^d^	Carboplatin_NANO_^d^
A2780	0.90 ± 0.06	0.75 ± 0.09	9 ± 1	11.2 ± 0.7
A2780cis	1.9 ± 0.2	1.5 ± 0.3	16 ± 1	15 ± 3
MCF-7	8 ± 2	8 ± 1	60 ± 5	65 ± 4
MDA-MB-231	2.6 ± 0.1	2.2 ± 0.4	30 ± 3	82 ± 7
MRC5 pd30	>100	>100	>100	>100

^a^The cells were incubated with the OLICARB nanocapsules for 72 h.

^b^The results are expressed as mean values ± SD of three independent experiments, each made in triplicate. The IC50 values are related to the concentration of carboplatin.

^c^Nanoparticles containing combinations of olaparib and carboplatin at the ratio of their molar concentrations of 1:1 (OLICARB_1:1_) or 2:1 (OLICARB_2:1_).

^d^Nanoparticles containing olaparib or carboplatin encapsulated as a single agent.

We also investigated cytotoxicity of nanoparticles containing olaparib or carboplatin encapsulated as a single agent. As shown in Table 2, the olaparib and carboplatin encapsulated as a single agent were markedly less cytotoxic than OLICARB nanoparticles in all cancer cell lines. It was verified that empty liposomes did not affect the viability of any cell line tested in the present work.

Apart from the ability of OLICARB nanoparticles to affect tumor cells resistant to clinically used cisplatin, OLICARB nanoparticles exhibit very low toxicity in noncancerous human cells derived from normal lung tissue (MRC5 pd30) (Table 2). The IC_50_ values of the OLICARB nanoparticles determined for noncancerous MRC5 pd30 cells were more than two orders of magnitude greater than those found for the sensitive cancer cell line A2780. Thus, the OLICARB nanoparticles show selectivity towards tumor cells relative to nontumorigenic normal cells so that it is conceivable that they may be recognized as a promising approach to improving the therapeutic index of anticancer agents.

### Localization in cancer cells by fluorescence confocal microscopy

**Figure 2:**
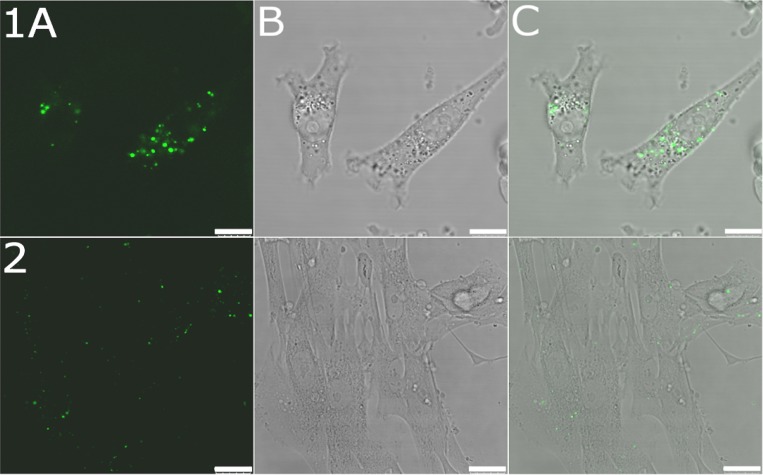
Confocal microphotographs of MDA-MB-231 and MRC-5 pd30 cells MDA-MB-231 cells (upper panel 1) and MRC-5 pd30 (bottom panel 2) were treated for 5 h with the OLICARB_1:1_ nanoparticles fluorescently labeled by encapsulation of 5-carboxyfluorescein; the final concentration of platinum and olaparib in the CF-labelled OLICARB nanocapsules was 1 µM, and that of CF was 0.1 µM. (**A**) Fluorescence channel. (**B**) Bright field. (**C**) Overlay of the fluorescence and bright field channels. Scale bar represents 10 μm for panel 1 and 25 μm for panel 2.

We have investigated the localization in MDA-MB-231 or non-malignant MRC-5 pd30 cells of the fluorescently labeled OLICARB nanoparticles with encapsulated 5-carboxyfluorescein (CF) in their lumen. The cells were treated with the CF-labelled OLICARB_1:1_ nanocapsules for 5 h and visualized by using confocal microscopy (Figure 2). No fluorescent signal was yielded by the untreated cells (not shown), cells treated with free (nonencapsulated) CF (0.1 µM) or empty nanocapsules (Supplementary Figure 2B) or OLICARB nanoparticles without encapsulated CF (not shown).

This observation suggests that the signal yielded by the cells treated with the OLICARB_1:1_ nanoparticles with encapsulated CF came only from the fluorescent dye transported into the cells in the intact nanoparticles. Figure 2 (upper panels 1) shows that most of the fluorescence signal was associated with cytoplasm, cytoplasmic membrane and probably endosomes of MDA-MB-231 cells confirming the cellular uptake of CF-labelled OLICARB nanocapsules. Figure 2 (bottom panels 2) also shows a similar pattern of the fluorescence signals associated with non-malignant MRC-5 pd30 cells treated with the fluorescently labeled OLICARB nanoparticles. The fluorescent signals were, however, markedly weaker very likely demonstrating a reduced accumulation of OLICARB nanoparticles in these cells.

The toxicities of the OLICARB nanocapsules and free (nonencapsulated) mixtures of the drugs (Tables 1 and 2) are different, and importantly, the cells treated with free (nonencapsulated) CF at the concentration corresponding to that of CF in the OLICARB nanoparticles yielded no fluorescent signal (Supplementary Figure 2, upper row A). Hence, it seems reasonable to suggest that a substantial fraction of our OLICARB nanocapsules enter the cells intact, still bearing both drugs (carboplatin and olaparib) and fluorescent dye CF, and that, very likely, intracellular accumulation of these nanoparticles is mediated mainly by endocytosis. The CF-labelled OLICARB nanocapsules have been shown to be distributed in the cytoplasm and effective to inhibit cell growth. Because carboplatin exerts its cytotoxic effects by binding to DNA in the nucleus [37] and olaparib acts as an inhibitor of the PARP [21], these observations suggest that the OLICARB nanoparticles subsequently decompose in the cytoplasm and carboplatin and olicarb are simultaneously released from the nanocapsules.

### Detection of apoptosis and necrosis

The levels of apoptosis and necrosis induced in MDA-MB-231 cells by free (nonencapsulated) carboplatin, olaparib, or the OLICARB_1:1_ nanocapsules at the equitoxic concentrations over 24 h exposure time were analyzed by flow cytometry. The cells were treated with the investigated agents and stained with annexin-V as the apoptosis marker together with propidium iodide as a necrosis marker. Flow cytometric analysis of MDA-MB-231 cells showed (Supplementary Figure 3) a clear direction of the treated cell populations through apoptosis (annexin-V positive) to double positive population (annexin-V and PI positive). The apoptotic mechanism of cell death seems to be similar for free carboplatin, olaparib, their respective nonencapsulated combination, and OLICARB_1:1_ nanocapsules. The minor population was only classified as a necrotic fraction (Supplementary Figure 3 and Supplementary Table 1).

### Quantification of the synergism observed for the co-treatment of cancer cells with the investigated agents

**Figure 3:**
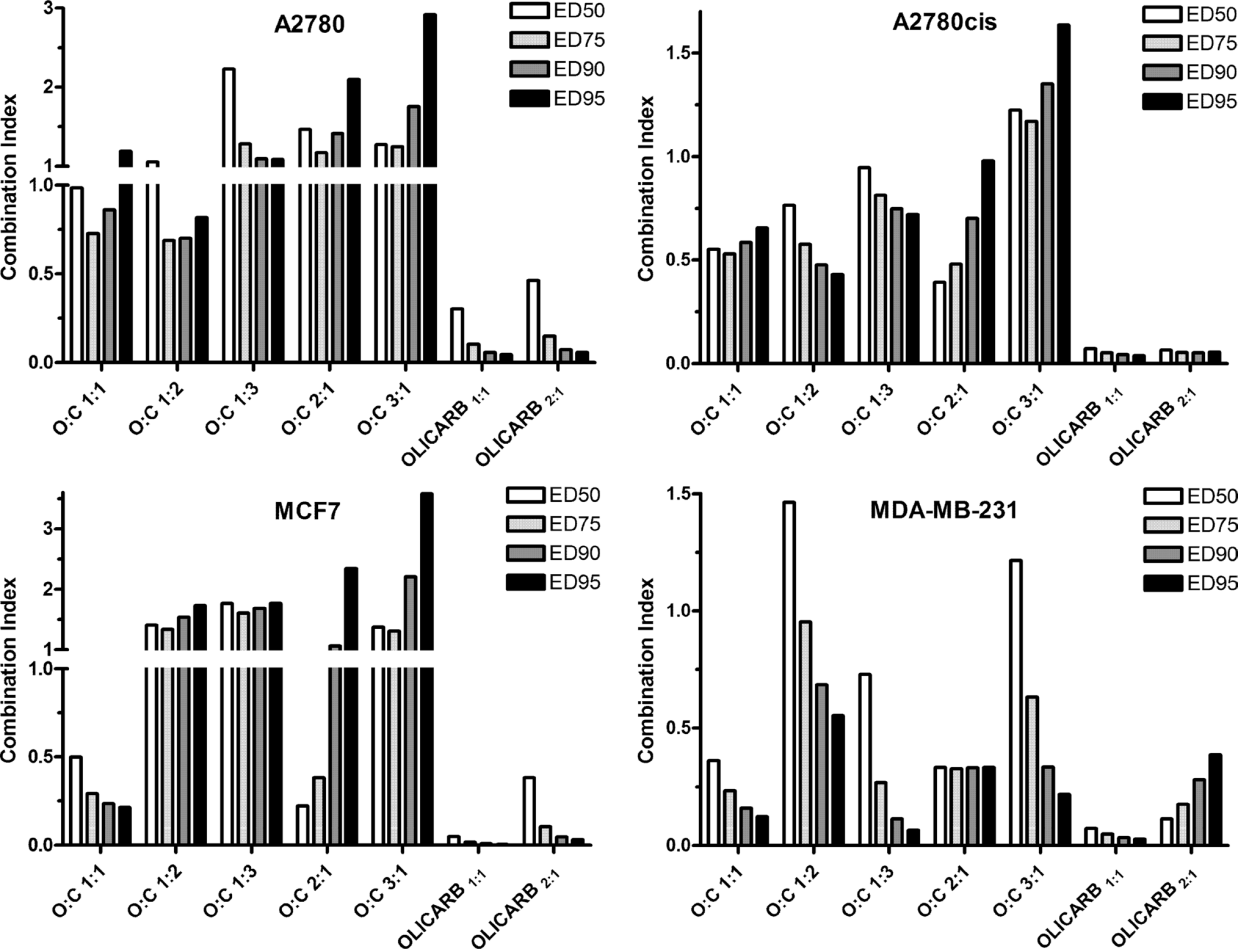
Combination index (CI) values obtained by analysis of the combinatorial effect of olaparib and carboplatin in the panel of human cancer cell lines The analysis was performed with CompuSyn software. The cells were incubated with the drugs for 72 h. The results are expressed as the mean values of three independent experiments, each made in quadruplicate. The SDs did not exceed 7% of the mean value.

It has been shown that the anticancer effect of platinum(II) anticancer drugs can be potentiated by PARP inhibitors including olaparib [9, 22–24, 27–29]. We used dose-inhibition studies to investigate the effects of mixtures of olaparib and carboplatin as free, single agents or encapsulated in the PEGylated liposomes (OLICARBs) at the ratios of their molar concentrations (olaparib:carboplatin) 1:1 or 2:1 in A2780, A2780cisR, MCF-7, and MDA-MB-231 cancer cells. The combination index (CI) method developed by Chou and Talalay [38, 39] was used to confirm and quantify the synergism observed for the co-treatment. The CI values were calculated using CompuSyn software, and these are summarized in Figure 3. CI < 1 is indicative of synergy. When CI > 1, antagonism is indicated, and a CI of approximately 1 is considered indicative of an additive response to the combination of drugs [40]. The values of CI were calculated for effective doses (ED) producing the 50, 75, 90 and 95% of the decrease in cellular viability.

Cotreatment of A2780 cells with nonencapsulated mixtures of olaparib and carboplatin [when the ratio of their molar concentrations (olaparib:carboplatin) was ≥1] caused a significant decrease in cell viability compared to the effects of free (nonencapsulated) carboplatin and olaparib used as single agents (Table 1). The data (Figure 3) obtained for the nonencapsulated mixtures of olaparib:carboplatin 1:1 and 1:2 indicated that carboplatin and olaparib were synergistic in A2780 cells or at least additive, whereas the combinations of the free drugs mixed at other ratios showed antagonism with CI > 1. A most pronounced synergism was observed in A2780cisR cells for all mixtures of nonencapsulated olaparib and carboplatin except for which a slight antagonism was found. In contrast, the data obtained for cotreatment with the nonencapsulated mixtures of olaparib and carboplatin indicated that carboplatin and olaparib were antagonistic or additive in MCF-7 cells, in particular when the data were evaluated for higher effective doses (ED_90_-_95_); the only exception was the cotreatment by the nonencapsulated mixture of olaparib:carboplatin of 1:1 when the data indicated a synergistic action of the two drugs. The greatest extent of synergy was observed for the cotreatment of MDA-MB-231 cells; only a slight antagonism was observed for the lowest effective dose (ED_50_) of the cotreatment with the nonencapsulated mixture of olaparib:carboplatin 2:1 and 3:1.

We also calculated the CI values for the combined treatment of cancer cells with the mixtures of olaparib:carboplatin 1:1 and 2:1 encapsulated in the PEGylated liposomes (OLICARB_1:1_ and OLICARB_2:1_). A great extent of synergism was found for the treatment of all cancer cell lines tested in the present work and all EDs (Figure 3). The values of CI ranged from 0.005 found for OLICARB_1:1_ at ED_95_ in MCF-7 cells up to 0.46 found for OLICARB_2:1_ at ED_50_ in A2780 cells. This result was not surprising due to the very high antiproliferative effects of the OLICARB nanoparticles (Table 2) and the rational design of the drug combinations for encapsulation.

### The assessment of DNA damage in cancerous cells treated with the investigated compounds using immunofluorescence analysis of γH2AX expression

The results demonstrating that olaparib and carboplatin were synergistic in several cancer cell lines also suggest that these drugs manifest their toxic effects in cancer cells by a molecular mechanism involving the same target. Antitumor effects of platinum(II) anticancer drugs, such as carboplatin, are due to inhibition of DNA and RNA polymerization by the adducts of these drugs formed on template DNA. Olaparib acts as an efficient PARP inhibitor preventing the repair of DNA damage. Hence, the augmentation of the toxic effects of carboplatin by olaparib in cancer cells could be a consequence of the inhibition of repair of platinated DNA leading to an accumulation of cytotoxic lesions in DNA [41].

To investigate this possibility, we investigated the extent of DNA damage caused by the combined action of olaparib and carboplatin, free or encapsulated in the OLICARB nanoparticles, in cancerous MDA-MB-231 cells. We used an immunofluorescence-based assay employing γ-H2AX as a biomarker of DNA damage in cells [42]. This very sensitive and reliable method allows the visualization of discrete nuclear foci formed as a result of H2AX phosphorylation. γ-H2AX was originally identified as an early event after the direct formation of DNA double-strand breaks (DSBs) [43]. However, DSBs are also formed indirectly by the collision of replication forks at sites of DNA damage, including DNA adducts and due to the repair of DNA damage [44]. Interestingly, it has been shown that platinum(II) anticancer drugs generate phosphorylated histone H2AX (γH2AX) foci through DNA platination at damaged replication forks [41, 45, 46].

**Figure 4:**
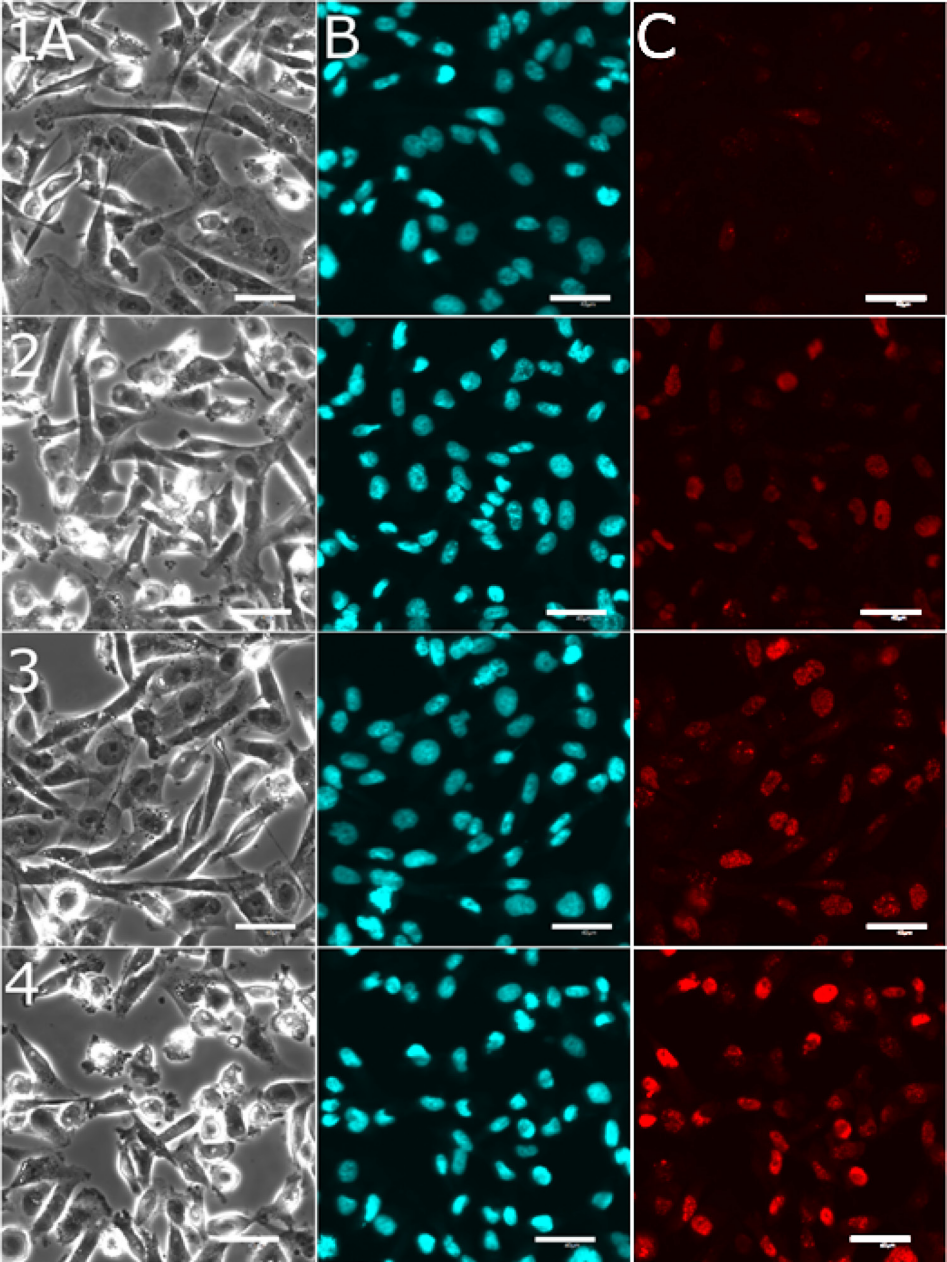
Representative confocal microscopic images recorded for detection of DNA damage in cancerous MDA-MB-231 cells The cells were treated with the investigated compounds by immunofluorescence based assay employing γ-H2AX. MDA-MB-231 cells untreated (row 1) or after 24 h of treatment with nonencapsulated carboplatin (151 µM, row 2); nonencapsulated olaparib (37 µM, row 3); OLICARB_1:1_ (concentration of carboplatin or olaparib was 2.6 µM, row 4) at 37° C and subsequent staining with Hoechst 33342 dye and γ-H2AX antibody. Channels (**A**) bright field; (**B**) nucleus visualized with Hoechst 33342 dye; (**C**) red fluorescence shows the sites (foci) of H2AX phosphorylation, which correspond to DNA damage (detected using γ-H2AX antibody). Scale bars represent 40 μm.

**Figure 5:**
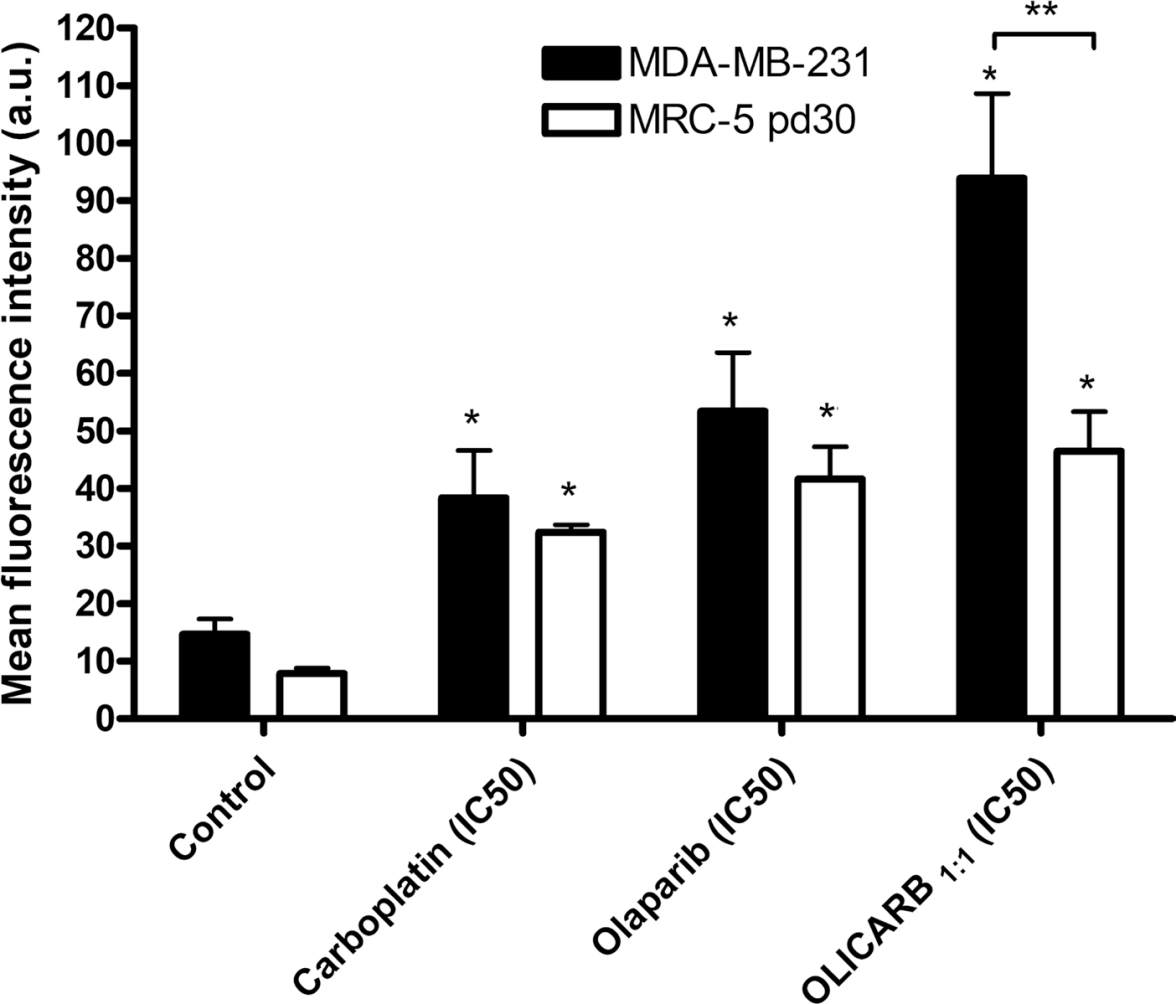
Quantification of DNA damage in cancerous MDA-MB-231 and noncancerous MRC pD30 cells The cells were treated with carboplatin, olaparib used as nonencapsulated single agents and their combination encapsulated in OLICARB_1:1_ by immunofluorescence-based assay employing γ-H2AX. For other details, see Figures 4 and S5. Analysis of the mean nuclear fluorescence intensity of γ-H2AX. Error bars indicate the SDs, statistical analysis was calculated with non-parametric students´ *t*-test; the symbol (^*^) denotes a significant difference (*p* < 0.01) from the untreated control; the symbol (^**^) denotes a significant difference (*p* < 0.001) of the mean fluorescence intensity observed for MDA-MB-231 and MRC-5 pd30 cells. Data are the mean ± SD obtained from at least three different experiments each performed in triplicate with at least one hundred cells per analysis.

MDA-MB-231 cells were treated for 24 h at 37° C with the investigated agents and analyzed using confocal microscopic detection for total γ-H2AX nuclear fluorescence (Figures 4 and 5). The cells were co-stained with Hoechst 33342 to confirm the co-localization of the detected fluorescence with the cell nucleus (Figure 4). In these experiments, the cells were treated with the investigated agents at the equitoxic concentrations corresponding to their IC_50_ values determined for the treatment lasting 72 h (Table 1). γ-H2AX nuclear fluorescence increased after the treatment of the cells with the compounds used as nonencapsulated single agents, namely carboplatin 2.6-fold or olaparib 3.6-fold compared with the untreated cells. The cells were also treated with the OLICARB_1:1_ nanoparticles and in this case γ-H2AX nuclear fluorescence increased even 6.2-fold compared with the untreated cells.

In agreement with the cytotoxic experiments (Tables 1 and 2), the synergistic effects of both drugs positively correlated with a significant increase in DNA damage. When comparing the data, it is evident that the combination of both drugs in the liposomes induced a higher proportion of DNA damage than both drugs used as nonencapsulated single agents.

For comparative purposes, we also assessed DNA damage using immunofluorescence analysis of γH2AX expression also in non-cancerous cells MRC5 pd30 treated with the investigated compounds (Figure 5 and Supplementary Figure 4). This result shows that the fluorescence associated with γH2AX expression was markedly lower for noncancerous MRC-5 pD30 than for cancerous MDA-MB-231 cells although a markedly higher concentration of the OLICARB was used in the case of the treatment of the noncancerous cells (152 µM vs. 2.6 µM).

### Activity of PARP in MDA-MB-231 cells

**Figure 6:**
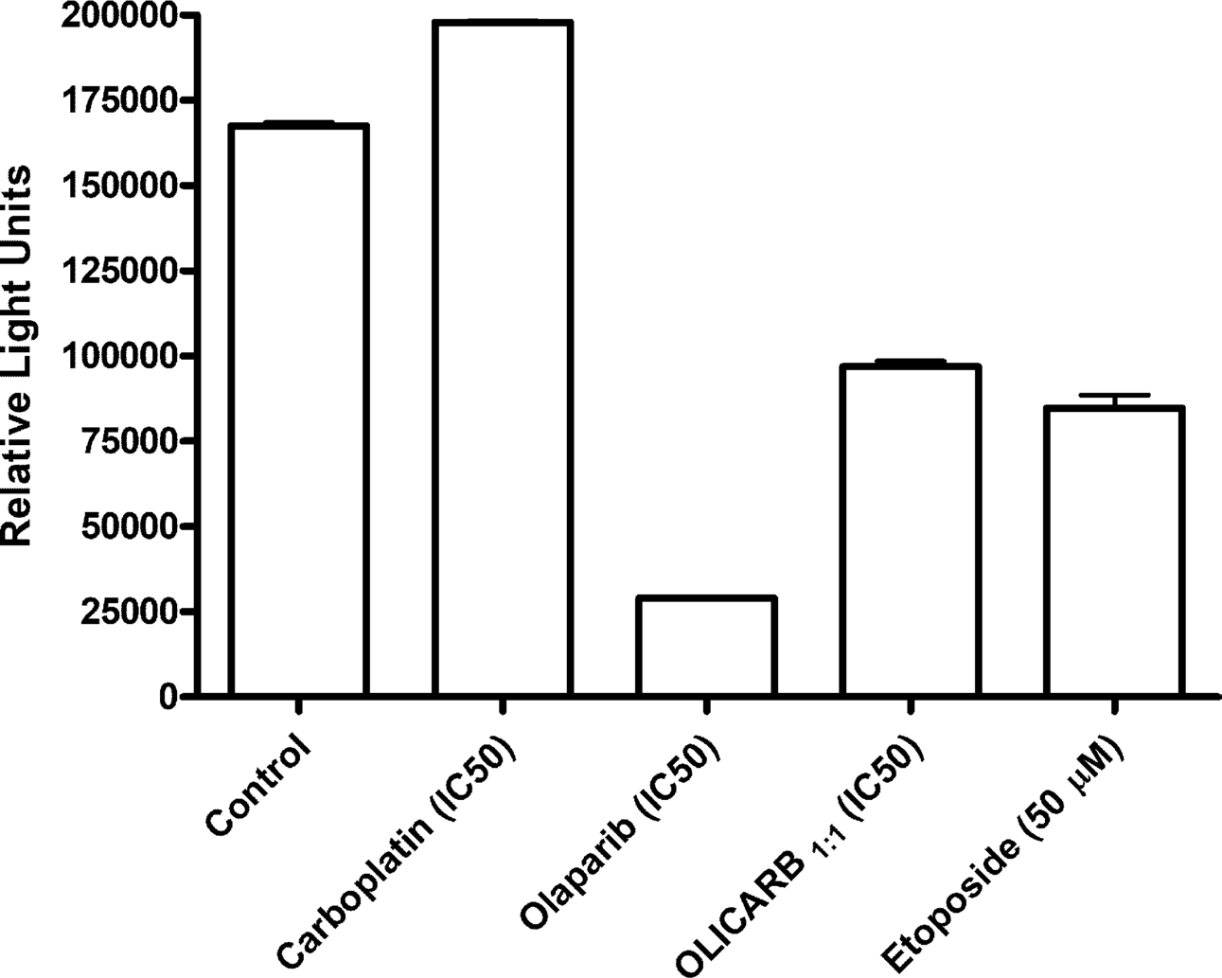
The activity of PARP in MDA-MB-231 cells Cells were exposed for 24 h to equitoxic concentrations of the investigated compounds (IC_50_,72 h) or etoposide (50 μM). Data are the mean ± SD from three independent experiments, each performed in quadruplicate.

Activity/inhibition of the PARP enzyme was detected using HT chemiluminescent PARP/Apoptosis assay. MDA-MB-231 cells were treated with the equitoxic concentrations of the investigated compounds (IC_50, 72 h_) for 24 h, and the cell lysates were analyzed for their PARP activity (Figure 6). A somewhat higher activity than that observed for the control sample was observed after the treatment with free (nonencapsulated) carboplatin. On the other hand, the lowest activity of the PARP enzyme was detected in the cells treated with free olaparib. The OLICARB_1:1_ also considerably decreased the activity of the PARP enzyme, although less than nonencapsulated olaparib. The data indicates that free olaparib can effectively compromise the tendency of carboplatin to stimulate the activity of PARP. Collectively, the mechanism of action of the combination of carboplatin and olaparib encapsulated in the OLICARB nanoparticles appears to involve the DNA damage by carboplatin augmented by the synergistic action of olaparib as an effective PARP inhibitor.

### Cytotoxicity testing in three-dimensional (3D) spheroid cell culture

In preclinical research of antitumor drugs, their testing using two-dimensional (2D) cell cultures still preponderates. However, the use of 2D cultures is connected with serious limitations because 2D cell monolayers represent an environment, distinctly different from that in native tumors in which most of the tissues are 3D [47, 48]. Cells grow in complex 3D cultures with heterogeneous regions, nutrient and oxygen gradients, intercellular and cell-extracellular matrix interactions which more closely reflect the tumor microenvironment [49]. Therefore, in this study, we investigated the effect of olaparib, carboplatin, their combinations and OLICARB nanoparticles under 3D cell culture conditions to provide more relevant data on their antitumor activity.

**Figure 7:**
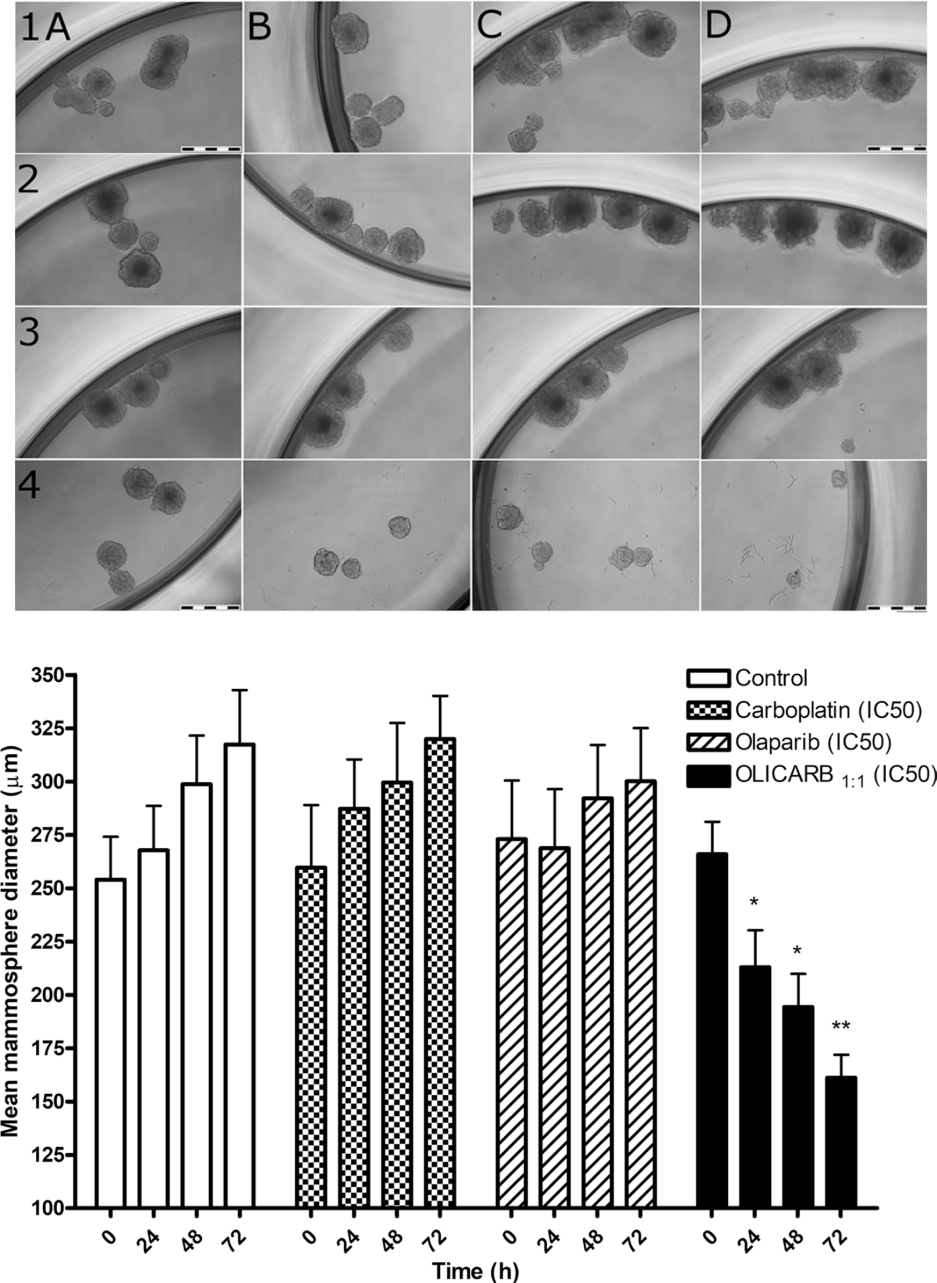
Representative bright-field images of the mammospheres formed with MCF-7 cells untreated and treated with the investigated compounds The cells were treated with the investigated compounds at their respective IC_50_ values [determined for the treatment lasting 72 h (Table 1)] if not stated otherwise. Upper panel: Rows: 1, control, untreated cells; 2–4, the cells treated with carboplatin (2); olaparib (3); OLICARB_1:1_ (4) for 0 h (column A), 24 h (column B), 48 h (columns C) and 72 h (column D). Cells were seeded in quadruplicate. Scale bars represent 500 μm. Lower panel: Quantification of mammosphere formation with MCF-7 cells untreated and treated with the investigated compounds for 24, 48 and 72 h at their respective IC_50_ values [determined for the treatment lasting 72 h (Table 1)]. Error bars indicate the SDs, statisticalanalysis was calculated with one-way ANOVA and non-parametric students´ *t*-tests; the symbols ^*^ and ^**^ denote a significant difference (*p* < 0.05 and *p* < 0.001, respectively) from the sham treated control. Data are the mean ± SD obtained from at least three different experiments each performed in triplicate.

The assay based on the reduction of tumor spheroid growth was used to assess the ability of free (nonencapsulated) carboplatin, olaparib, and OLICARB nanoparticles (at their respective IC_50_ values determined after 72 h treatment) to inhibit 3D mammosphere formation from MCF-7 single-cell suspensions. The 3D mammospheres were cultured for 96 h to grow up to the tissue mass of around 250 μm in diameter as described in the Materials and methods. The cells were treated with the investigated compounds, visualized every 24 h of the treatment and analyzed for their sphere mass (Figure 7). The control cells (treated with the empty liposomes) showed a continual increase of solid mass characterized by the growth of sphere diameter by approximately 21 μm every 24 h. Roughly the same effect was observed when the mammospheres were treated with free (nonencapsulated) carboplatin (20 μm increase every 24 h). The free (nonencapsulated) olaparib stopped the sphere growth in the first 24 h and somewhat suspended the growth of spheres during additional 48 h of the treatment. The OLICARB_1:1_ nanoparticles showed a pronounced inhibitory effect on the breast spheres, the size of the spheroids decreased every 24 h by approximately 35 μm; the microscopic studies also showed that some spheres were fully broken and dissociated. Collectively, these data confirm that the encapsulation of carboplatin and olaparib into liposomal constructs to form the OLICARB nanoparticles is the viable approach for the treatment of solid-mass breast tumors with the aim to eliminate the possible effects of acquired resistance.

## MATERIALS AND METHODS

### Materials

Carboplatin (purity 99.91%) and olaparib (purity 99.83%) were from Selleck Chemicals LLC (Houston, USA). Phospholipids for the preparation of nanocapsules were from Avanti Polar Lipids (Birmingham, Alabama, USA). The human ovarian carcinoma cell lines A2780 (cisplatin-sensitive expressing wild-type p53, BRCA1 and BRCA2 [58]), A2780cisR (variant of A2780 cells with acquired resistance to cisplatin [59, 60] expressing wild type BRCA1 [61]), the human breast adenocarcinoma cells MCF-7 [59, 60] expressing wild type BRCA1 and BRCA2 [62–64] and the human MRC-5 pd30 cell line with wild type BRCA1 and BRCA2 [65, 66] derived from normal (noncancerous) lung tissue were obtained from the European collection of cell cultures (ECACC) (Salisbury, UK). The human breast carcinoma MDA-MB-231 cell line (derived from the metastatic site [59, 60] expressing wild type BRCA1 and BRCA2 [62–64, 67]) was obtained from ATCC (University Blvd. Manassas, USA). RPMI 1640 medium, fetal bovine serum (FBS), and Dulbecco’s modified eagle’s medium (DMEM) were obtained from PAA (Pasching, Austria). RPMI 1640 medium was supplemented with 10% heat-inactivated FBS and gentamycin (50 μg mL^-1^, Serva, Heidelberg, Germany). The acquired resistance of A2780cisR cells was maintained by supplementing the medium with cisplatin (1 μM) every second passage. The cells were cultured in a humidified incubator at 37° C, 5% CO_2_ atmosphere and subcultured two times a week with an appropriate plating density. 3-(4,5-Dimethyl-2-thiazolyl)-2,5-diphenyl-2H-tetrazolium bromide (MTT) was obtained from Calbiochem (Darmstadt, Germany).

### Preparation and characterization of nanoparticles containing combinations of olaparib and carboplatin (OLICARB)

Liposomal nanoparticles (OLICARB) were prepared according to the standard procedure for the preparation of cisplatin nanocapsules by repeatedly freezing and thawing concentrated solutions containing mixtures of olaparib and carboplatin in the presence of negatively charged phospholipids (Avanti Polar Lipids, Birmingham, Alabama, USA), cholesterol and polyethyleneglycol [68]. This procedure was used with the following slight modifications: Nanoparticles containing combination of olaparib and carboplatin at the ratio of their molar concentrations of 2:1 (OLICARB_2:1_) were prepared by mixing carboplatin (3.3 mM) dissolved in H_2_O with olaparib (4.95 mM) dissolved in dimethylsulfoxide (DMSO) (3%); this mixture was used to hydrate a lyophilized phospholipid film consisting of a mixture of 1,2-dioleoyl-*sn*-glycero-3-phosphocholine, 1,2-dioleoyl-*sn*-glycero-3-phosphoserine, and cholesterol in the ratio of 3:3:4 plus 1,2-distearoyl-sn-glycero-3-phosphoethanolamine-N-[amino(polyethylene glycol)-2000] (PEG, 6%). Nanoparticles containing a combination of olaparib and carboplatin at the ratio of their molar concentrations of 1:1 (OLICARB_1:1_) were prepared in the same way, except that the concentration of carboplatin was 6.6 mM. These lipid dispersions (consisting of 1.2 mM phospholipid) (OLICARB_2:1_ and OLICARB_1:1_) incubated at 37° C for 60 min were subsequently subjected to 10 freeze–thaw cycles by using ethanol/dry ice (−70° C) and an Eppendorf Thermo-Mixer F1.5 instrument (Eppendorf AG, Hamburg, Germany; 37° C). The resulting colloidal solutions were transferred to microfuge tubes and centrifuged for 10 min at 15 000 rpm at 4° C in an MSE Hawk 15/05 microcentrifuge (Henderson Biomedical Ltd., London, UK) to collect the nanocapsules. After removal of the supernatant, the fluffy white layer on top of the white pellet, corresponding to large liposomes, was removed by using a micropipette. The white pellet containing the OLICARB nanocapsules was resuspended in water (1 mL) and centrifuged three times, to wash away nonencapsulated olaparib and carboplatin. Nanocapsules containing only carboplatin or olaparib were prepared by the same way as the OLICARB nanoparticles, but the encapsulation solution contained only the single drug (carboplatin or olaparib). Upon resuspending the final pellet in water (0.5 mL), the nanocapsules were stored at 4° C. Nanocapsules with a narrower size distribution were obtained by high-pressure extrusion through Nucleopore polycarbonate membranes (Whatman International Ltd., Avanti Polar Lipids, USA) with 100 nm pore sizes. The empty nanoparticles were prepared in parallel and were used as the negative controls. Hydrodynamic diameter (*d*_H_) and zeta potential values (ξ_P_) were determined using a Malvern Zetasizer Nano instrument (Malvern Instruments Ltd., Worcestershire, UK). The phospholipid content of the nanocapsules was determined after aliquots of the sample (0.05 mL) were digested with 2 mL of HNO_3_ (Merck, p.a.) and 0.2 mL of H_2_O_2_ (Merck, p.a.) in a PFA (perfluoroalkoxy) beaker on a hot plate. The cooled digested samples were filled up to 50 mL by deionized water. The analyses of P and Pt were performed by ICP-MS. The platinum content in the nanoparticles was also determined by ICP-MS, and the concentration of olaparib in the nanoparticles was determined by UV absorbance spectrophotometry (olaparib showed characteristic absorption peaks at 276, 290, 299 and 311 nm and the corresponding extinction coefficients were 8432, 5926, 5096 and 3548 Lmol^-1^cm^-1^ respectively). Electron microscopy images were obtained by using a transmission electron microscope (TEM JEM2010 microscope) operated at 200 kV with a point-to-point resolution of 1.9 Å.

### Release of carboplatin and olaparib from OLICARB nanocapsules

Suspensions of OLICARB nanocapsules (200 µL) were incubated in the DMEM medium (10% FBS, 50 μg mL^-1^ gentamycin, pH 6.8 or 7.4) at 4° C or 37° C. At the predetermined times (0.5, 3, 7, 12, 24, 96 and 120 h), the aliquots were withdrawn and centrifuged (20 000 g, 5 min). Platinum or olaparib content in the supernatant was determined by FAAS or UV absorption spectrophotometry, respectively.

### *In vitro* growth inhibition assay

The effects of OLICARB nanoparticles, free (nonencapsulated) carboplatin, free olaparib, and the defined combinations (mixtures) of both free compounds on the viability of malignant and nonmalignant cells were tested by using the colorimetric MTT assay. Cells were seeded in 96-well tissue culture plates according to their growth profiles at the densities necessary to obtain 90–95% confluence in the controls at the end of the treatment (96 h post-seeding) and incubated at 37° C in a humidified 5% CO_2_ atmosphere overnight (16 h). Subsequently, the cells were treated with the nanocapsules, free carboplatin, olaparib or with their combinations and incubated for an additional 72 h. Empty liposomes were tested in parallel. MTT solution (10 µL, 5 mg mL^-1^) was added to each well, and the plates were incubated for 4 h. At the end of the incubation time, the medium was removed, and the formazan product was dissolved in 100 mL of DMSO per well. Cell viability was evaluated by measuring the absorbance at λ = 570 nm by using a Sunrise Tecan Schoeller absorbance reader. Cytotoxic effects were expressed as IC_50_ values, which were related to the concentration of platinum. The IC_50_ values were calculated from curves constructed by plotting cell survival (%) versus platinum concentration (µM). All experiments were performed at least in triplicate. The reading values were converted into the percentage of the control (% cell survival). The concentrations of platinum were checked by FAAS to verify the real concentration of carboplatin in the culture medium during the treatment.

### Cellular localization of OLICARB nanocapsules fluorescently labeled with 5-carboxyfluorescein (CF)

The fluorescently labeled OLICARB nanocapsules were prepared using the standard procedure with some modifications. Briefly, dry lipid films were hydrated with carboplatin (3.3 mM) dissolved in H_2_O, olaparib (4.95 mM) dissolved in DMSO and CF (0.3 mM) dissolved in DMF. The fluorescence spectrum of the fluorescently labeled OLICARB is shown in Supplementary Figure 5. MDA-MB-231 and MRC-5 pd30 cells were seeded on glass bottom dishes (P35G-0-14-C, MatTek Co., Ashland, USA) at the density of 3 × 10^5^ cells per dish, cultured in DMEM medium (without phenol red) and incubated at 37° C in a humidified 5% CO_2_ atmosphere. After the overnight incubation, the cells were treated with the CF-labelled OLICARB or nonencapsuled CF. The final concentration of platinum and olaparib in the CF-labelled OLICARB nanocapsules was 1 µM, and that of CF was 0.1 µM. After the 5 h incubation period, cells were washed with PBS and resuspended in a fresh medium. Samples were visualized with a Leica TSC SP8 SMD laser scanning confocal microscope (Leica Microsystems, Wetzlar, Germany). Excitation/emission wavelengths were 492/517 nm.

### Flow cytometric analysis of the cell death

The MDA-MB-231 cells were seeded in 100 mm tissue culture dishes at a density of 1·10^6^ cells per dish. After overnight incubation, the cells were treated with OLICARB_1:1_, free carboplatin and olaparib for 24 h at the concentrations corresponding to their IC_50_ values determined after 72 h-treatment. The negative controls (untreated cells) were also analyzed. Following the incubation, the cells were collected, and aliquots of 0.5·10^6^ cells were resuspended in 100 µL of annexin-V binding buffer (10 mM HEPES, 0.14 M NaCl, 2.5 mM CaCl_2_, pH 7.4). Subsequently, the cells were stained with annexin-V-pacific blue conjugate (Exc. 410 nm/Em. 455 nm; 5% v/v) (Invitrogen, USA) and propidium iodide (Exc. 535 nm/Em. 617 nm; 10 µg mL^-1^) (≥94%; HPLC, Sigma, Czech Republic) and incubated for 15 min at room temperature. Cells were analyzed immediately by flow cytometry (BD FACSVerse), and the data were analyzed using BD FACSSuite software. 3·10^4^ events were analyzed; the dot plots are the representatives of three independent experiments.

### Determination of drug synergy

A2780, A2780cisR, MCF-7, and MDA-MB-231 were seeded in quadruplicate in 96-well plates, incubated overnight and treated with the investigated agents at the indicated doses for another 72 h. Growth inhibition was determined using an MTT assay. The data were analyzed using CompuSyn program (Combosyn, Inc., Paramus, NJ, USA) and calculated from the mean values of four independent experiments. Drug synergy was determined by the CI method (CI = combination index) [40]. CIs were calculated at ED_50_, ED_75_, ED_90_, ED_95_ (ED = effective dose, ED_XY_ is the concentration of the drug that produces a quantal effect (all or nothing) in XY% of the cell population).

### Immunofluorescence analysis of formation of γH2AX foci

For γH2AX staining, MDA-MB-231 and MRC-5 pd30 cells were seeded on 35 mm glass bottom confocal dishes (MatTek Co, Ashland, USA) at the density of 0.6 × 10^6^ cells/dish. After overnight incubation, the cells were treated for 24 h at 37° C with the compounds tested at the concentrations corresponding to the IC_50_ values (related to the concentration of platinum) determined for the treatment lasting 72 h; the cells were treated with the OLICARB_1:1_ nanoparticles also at the concentration corresponding to the IC_50_ value determined for combination of the nonencapsulated carboplatin+olaparib (C:O = 1:1). γ-H2AX was determined using the HCS DNA Damage kit (Invitrogen – Molecular Probes, Eugene, USA). After the treatment, the cells were washed with phosphate buffered saline (PBS), fixed using 4% formaldehyde solution for 15 min at 25° C washed again with PBS and the cell membranes were permeabilized with 0.25% Triton-X100 PBS solution for 15 min at 25° C to facilitate antibody penetration. The cells were washed again with PBS and incubated for 60 min at 25° C with the blocking solution 1% (w/v) bovine serum albumin. Subsequently, the cells were incubated with primary γH2AX antibody for 60 min at 25° C, washed three times with PBS incubated/counterstained with secondary goat anti-mouse IgG (H+L) Alexa Fluor^®^ 555 antibody and Hoechst 33342 dye for another 60 min at 25° C. At the end of the procedure, the cells were washed, and the dish was filled with PBS. Samples were visualized by confocal microscopy using Olympus FV10i microscope, and the images were analyzed with ImageJ software (National Institute of Health, Bethesda, MD, USA). For quantification of γH2AX foci, at least 100 cells from each group were visually scored. Cells showing more than 10 foci were counted as positive for γH2AX. The images were obtained for the samples obtained from at least three independent experiments.

### Activity of PARP in MDA-MB-231 cells

The MDA-MB-231 cells were seeded in 96-well tissue culture plates at the density of 5·103 cells/well. After the overnight incubation, the cells were treated with the equitoxic concentrations of the investigated compounds corresponding to their IC_50_ values determined after 72-h treatment and etoposide at the concentration of 50 μM was included as the positive control for the PARP inhibition. Following the 24 h incubation period, 300 ng of protein from cell lysates was transferred to histone coated well plates included in HT chemiluminescent PARP/Apoptosis assay (Trevigen^®^, MD, USA). The manufacturers´ instructions recommended for this assay were followed in the subsequent steps. The chemiluminescent signal was detected on Infinite200 (Tecan).

### Generation of spheroids and analysis of their growth

The human breast Caucasian adenocarcinoma derived from the pleural effusion MCF-7 cells (ECACC lot#13K023) was used. The cells were transferred to 3D forming conditions on ultra-low attachment (ULA) plastics (Corning, NY, USA). The cells were seeded as the single cells on 96-well ULA plate and cultured in DMEM medium enriched with 2% B27 supplement (Thermo Fisher Scientific Inc., MA, USA), 20 ng mL^-1^ epidermal growth factor (EGF; Sigma Aldrich, Darmstadt, Germany) and 0.15% (w/v) human serum albumin (Sigma Aldrich, Darmstadt, Germany) and incubated for 96 h to initiate the formation of mammospheres. Subsequently, the spheroids with the mean diameter of about 250 μm were treated for 24–72 h at 37° C with the compounds tested at the concentrations corresponding to the IC_50_ values (related to the concentration of platinum) determined for the treatment lasting 72 h; the cells were treated with the OLICARB_1:1_ nanoparticles also at the concentration corresponding to the IC_50_ value determined for a combination of the nonencapsulated olaparib + carboplatin 1:1. Samples were photographed for the analysis of the morphology of spheroids, visual scoring and analyzed for their diameter every 24 h after the treatment commenced until 72 h. It was verified that the empty liposomes had no impact on the mammosphere characteristics relative to the untreated controls. Samples were photographed by using Canon EOS 1200D camera attached to Olympus CKX41 inverted microscope with 10×/0.25 phase contrast objective. Digital images were acquired and analyzed by QuickPHOTO MICRO 3.1 program (PROMICRA, Prague, Czech Republic).

### Other physical methods

The platinum content was quantified by ICP-MS with an Agilent 7500 spectrometer (Agilent, Japan) or FAAS with a Varian AA240Z Zeeman atomic absorption spectrometer equipped with a GTA 120 graphite tube atomizer. If not stated otherwise, before analysis of the platinum content, the samples were digested in hydrochloric acid (11 M) by using the Microwave Accelerated System MARS5 (CEM, GmbH, Kamp-Lintfort, Germany). Electron microscopy was performed on TEM JEM2010 microscope. All the values are the means ± SD of not less than three independent experiments.

### Statistical analysis

Experimental data were subjected to statistical analysis using an analysis of variance (ANOVA) and non-parametric student´s *t*-test. If not mentioned otherwise, the symbol (^**^) denotes a significant difference (*p* < 0.001) from the untreated control; (^*^) denotes a significant difference (*p* < 0.01).

## CONCLUSIONS

At present, chemotherapy combining anticancer drugs is of great interest since it can maximize efficacy through broadening therapeutic index, minimizing the development of multidrug resistance and simultaneously reduce systemic toxicity due to the delivery of lower drug doses [50]. We present an approach for developing a delivery system for a combined anticancer chemotherapy involving carboplatin and olaparib. An advantage of this combination may comprise the potentiation of the efficacy of DNA damaging agent, carboplatin, by olaparib which acts as an efficient PARP inhibitor preventing the repair of DNA damage. The *in cellulo* data obtained for the combinations of olaparib plus carboplatin (Figure 3) indicate that the mixtures of olaparib and carboplatin are synergistic in ovarian tumor cells A2780 or triple negative breast cancer cells MDA-MB-231 when mixed in the ratio of 1:1 and 1:2. In contrast, they are antagonistic in luminal A breast cancer cells MCF-7. However, it was previously shown that the combinations administered as free drug cocktails rapidly distribute into healthy and tumor tissues at drug ratios that differ from those which were administered; this represents drawback of the combined anticancer chemotherapy. Early studies [51, 52] demonstrated that an optimal drug combination ratio *in vivo* could be maintained through encapsulation of the combined drugs in liposomes. Moreover, *in vitro* drug interaction effects could be translated *in vivo* since liposomes can synchronize pharmacokinetics and biodistribution of drug combinations and deliver them to tumor tissue at the specific drug ratio [53].

We show in the present work (Figure 3) that synergistic action of combinations of olaparib and carboplatin is considerably improved if these combined drugs are encapsulated into liposomes at the defined concentration ratios. Upon formation of such nano-formulations, called OLICARB, activity in human ovarian and breast cancer cell lines, including those with acquired or inherited resistance to platinum antitumor drugs used in the clinic, is still markedly improved. Notably, the OLICARB nanoparticles show marked selectivity towards tumor cells relative to nontumorigenic normal cells (Table 2) so that it is conceivable that they may be recognized as a promising approach to improving the therapeutic index of anticancer agents. In addition, the combination of both drugs embedded in the OLICARB nanoparticles induces a higher proportion of DNA damage in the cancer cells than both drugs used as single agents. These results are consistent with the view and support the hypothesis that the enhancement of the toxic effects of carboplatin by olaparib in cancer cells is a consequence of the inhibition of repair of platinated DNA leading to an accumulation of cytotoxic lesions in DNA.

To provide more relevant data on the antitumor activity of OLICARB nanoparticles, we also studied the effect of the investigated agents under 3D cell culture conditions. The results of these experiments (Figure 7) demonstrate that the combination of olaparib with carboplatin if encapsulated in the OLICARB nanoparticles is particularly effective to inhibit the growth of 3D mammospheres. Hence, these results reinforce our conclusion that the encapsulation of carboplatin and olaparib into liposomal constructs to form the OLICARB nanoparticles is the viable approach for the treatment of solid-mass breast tumors with the aim to eliminate the possible effects of acquired resistance.

We intended to show in this study that the encapsulation of the selected drug combination changes the cell response compared to the treatment with the non-encapsulated drugs. As the consequence of encapsulation, some drawbacks connected mainly with cell uptake and inactivation by nucleophiles in extracellular milieu are eliminated, and thus the behavior of the drug combination was changed after the encapsulation. The generally accepted mechanism of the cellular uptake of liposomes involves the adsorption of liposomes onto the cell surface and subsequent endocytosis [54]. Thus, any specific membrane transporters may play a role in the case of liposomal formulation compared to the non-encapsulated drugs [55, 56]. It is likely that also synergy of both drugs is different after the encapsulation. But, as we have shown, the ratio of olaparib: carboplatin in the OLICARB nanoparticles did not play so critical role as in the case of the non-encapsulated treatment schedule (compare Tables 1 and 2). Thus, the enhanced antitumor effect of olaparib and carboplatin used in combination and encapsulated in the OLICARB nanoparticles is caused mainly by the fact that both drugs are delivered simultaneously to cancer cells.

As a proof of concept, this report describes a strategy combining existing antitumor platinum-based drug and efficient, clinically approved PARP inhibitor to produce a well-defined system that considerably potentiates the toxic activity of the antitumor platinum drug used in the clinic in tumor cells. This system demonstrates advantages of liposomal drug combination delivery comprising mainly: (i) administration of combined drugs simultaneously; (ii) improved control of concentrations of drugs used in combination at the target sites by changing the ratio of combined drugs in liposomes; and (iii) achievement of maximal effects by synergistic action of the drugs used in combination after cellular uptake of liposomes [53, 57]. Thus, the findings presented in our study may have important implications for the assessment of OLICARB nanoparticles in further preclinical studies and the clinic.

## SUPPLEMENTARY MATERIALS FIGURES AND TABLE



## Abbreviations

Carboplatin: *cis*-diammine-[1,1-cyclobutanedicarboxylato]platinum(II)
CI: Combination index
Cisplatin: *cis*-diamminedichloridoplatinum(II)
DMEM: Dulbecco’s modified eagle’s medium
DMSO: Dimethylsulfoxid
ED: Effective dose
FAAS: Flameless atomic absorption spectrometry
FBS: Fetal bovine serum
IC_50_: Concentration of the agent inhibiting cell growth by 50%
ICP-MS: Inductively coupled plasma mass spectrometry
MTT: 3-(4,5-Dimethyl-2-thiazolyl)-2,5-diphenyl-2H-tetrazolium bromide
NER: Nucleotide excision repair
Olaparib: {4-[3-(4-cyclopropanecarbonylpiperazine-1-carbonyl)-4-fluorobenzyl]-2H-phthalazin-1-one}
PARP: P
PBS: phosphate buffered saline
PEG: 1,2-distearoyl-sn-glycero-3-phosphoethanolamine-N-[amino(polyethylene glycol)-2000]
SEM: Scanning electron microscope
