# Correction: Simultaneous delivery of olaparib and carboplatin in PEGylated liposomes imparts this drug combination hypersensitivity and selectivity for breast tumor cells

**DOI:** 10.18632/oncotarget.26304

**Published:** 2018-10-30

**Authors:** Vojtech Novohradsky, Juraj Zajac, Oldrich Vrana, Jana Kasparkova, Viktor Brabec

**Affiliations:** ^1^ Institute of Biophysics, Czech Academy of Sciences, Kralovopolska 135, CZ-61265 Brno, Czech Republic

**This article has been corrected:** The correct Grant information is given below:

**ACKNOWLEDGMENTS**

This work was supported by the Czech Science Foundation, Grant No. 18-09502S. The authors thank to Dr. Klára Čépe, Dr. Václav Ranc and Zuzana Chaloupková from the Regional Centre of Advanced Technologies and Materials (Olomouc, Czech Republic) for the TEM, DLS and ξ-potential analysis.

Original article: Oncotarget. 2018; 9:28456-28473. https://doi.org/10.18632/oncotarget.25466

