# Supplementary material for: Simultaneous delivery of olaparib and carboplatin in PEGylated liposomes imparts this drug combination hypersensitivity and selectivity for breast tumor cells
Source: Oncotarget. 2018 Jun 19;9(47):28456–73. doi: 10.18632/oncotarget.25466 (PMC6033346; doi:10.18632/oncotarget.25466)
Supplement: Supplementary file 1 [file oncotarget-09-28456-s001.pdf]

Simultaneous delivery of olaparib and carboplatin in PEGylated liposomes imparts this drug combination hypersensitivity and selectivity for breast tumor cells

SUPPLEMENTARY MATERIALS

Supplementary Table 1: Quantification of the flow cytometric data[a]

Quadrant/death event	Control	Carboplatin	Olaparib	$OLICARB_{1:1}$
UL (Nec+/Ap–)	0.8 ± 0.2	1.9 ± 0.1	1.1 ± 0.2	1.8 ± 0.3
UR (Nec+/Ap+)	1.4 ± 0.4	4.3 ± 0.4	3.6 ± 0.3	6.2 ± 0.7
LR (Nec–/Ap+)	1.7 ± 0.3	3.1 ± 0.2	4.1 ± 0.6	3.3 ± 0.5
LL (Nec–/Ap–)	96 ± 1	90.7 ± 0.7	91.2 ± 0.8	88.7 ± 0.4
Death - total (UL, UR, LR)	3.9 ± 0.6	9.3 ± 0.8	8.8 ± 0.9	11.3 ± 0.7

[a]Death events were categorized as a necrotic fraction (Nec) and apoptotic fraction (Ap) and qualified as positive (+) and/or negative (−). Total death events were summarized as the sum of the percentage in UL, UR and LR quadrants.

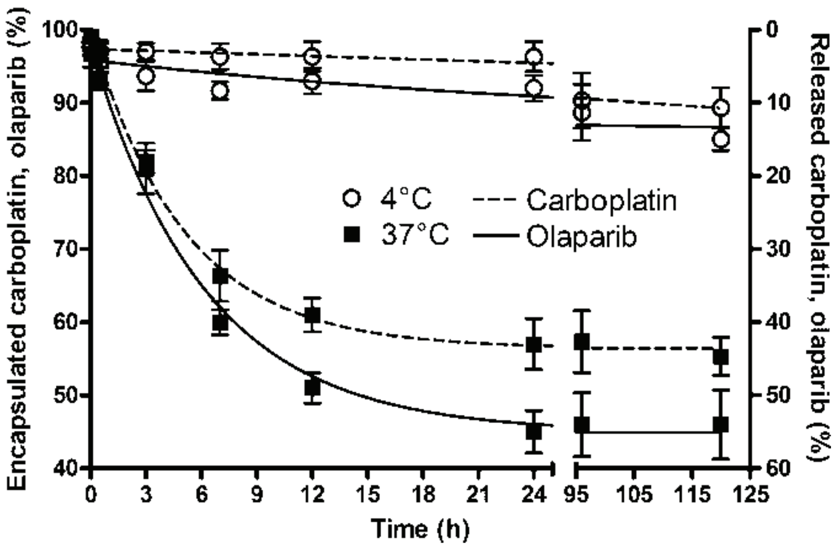

Supplementary Figure 1: Release kinetics of encapsulated compounds from OLICARB1:1. The release profile of carboplatin (dashed line) and olaparib (full line) from liposomes tested in the DMEM medium at 4° C and 37° C (pH 7.4).

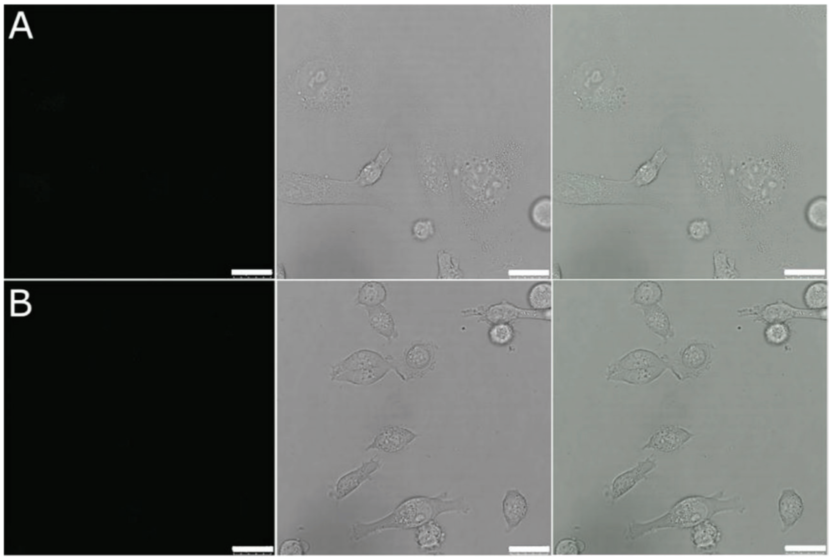

Supplementary Figure 2: Confocal microphotographs of MDA-MB-231 cells treated for 24 h with. (**A**) 0.1 μM 5-carboxyfluorescein (CF); the concentration of CF corresponded to that of CF contained in the OLICARB nanoparticles used in the experiments shown in Figure 2. (**B**) Empty nanocapsules. Left panels: Fluorescence channel. Panels in the middle: Bright field. Right panels: Overlay of the fluorescence and bright field channels. Scale bars represent 25 μm.

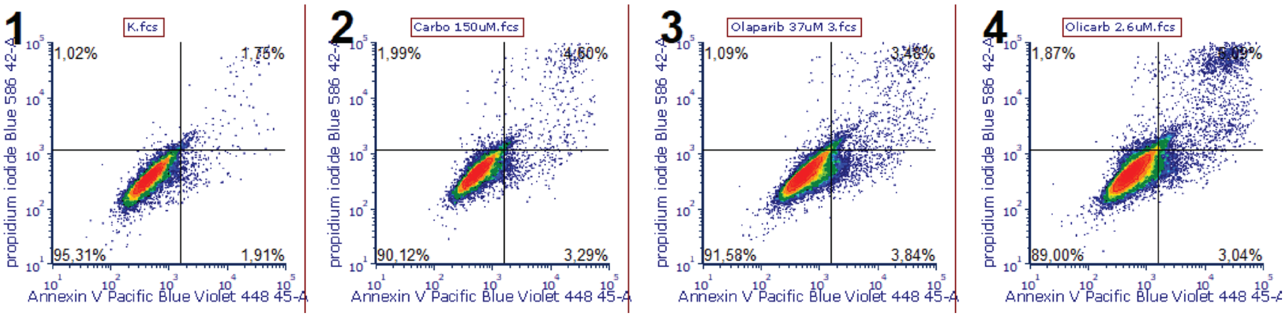

Supplementary Figure 3: Cell death analysis in MDA-MB-231 cells by flow cytometry. Cell death was analyzed using propidium iodide (necrotic population) and annexin-V-pacific blue conjugate(apoptotic population) after 24 h of the treatment. Cells were treated with the equitoxic concentrations of the investigated compounds corresponding to the IC50 values determined at 72 h. 1: Untreated control; 2: carboplatin (151 μM); 3: olaparib (37 μM) and 4: OLICARB1:1 (2.6 μM). Quadrants clockwise: upper left (UL) PI-positive/annexin-V-negative, upper right (UR) PI-positive/annexin-V-positive, low right (LR) PI-negative/annexin-V-positive and low left (LL) both annexin-V and PI negative. Thirty thousand events were analyzed, and the figures are representatives of three independent experiments.

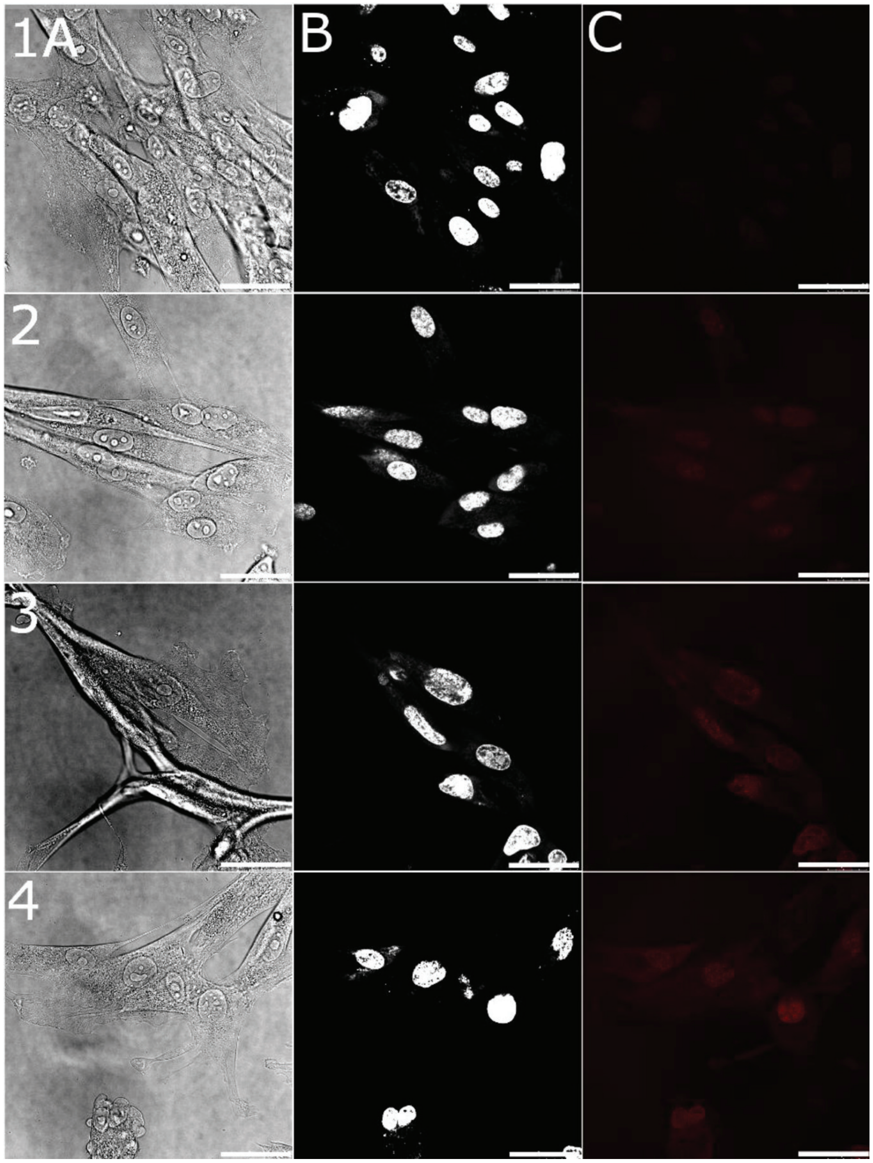

Supplementary Figure 4: Representative confocal microscopic images recorded for detection of DNA damage in non-cancerous MRC-5 pd30 cells. The cells were treated with the investigatedcompounds by immunofluorescence based assay employing γ-H2AX. MRC-5 pd30 cellsuntreated (left row 1) or after 24 h of treatment with nonencapsulated carboplatin (152 µM, left row 2); nonencapsulated olaparib (142 µM, left row 3); OLICARB1:1 (concentration of carboplatin or olaparib was 100 µM, right row 4) at 37° C and subsequent staining with Hoechst 33342 dye and γ-H2AX antibody. Channels (**A**) bright field; (**B**) nucleus visualized with Hoechst 33342 dye; (**C**) red fluorescence shows the sites (foci) of H2AX phosphorylation, which correspond to DNA damage (detected using γ-H2AX antibody). Scale bars represent 40 µm.

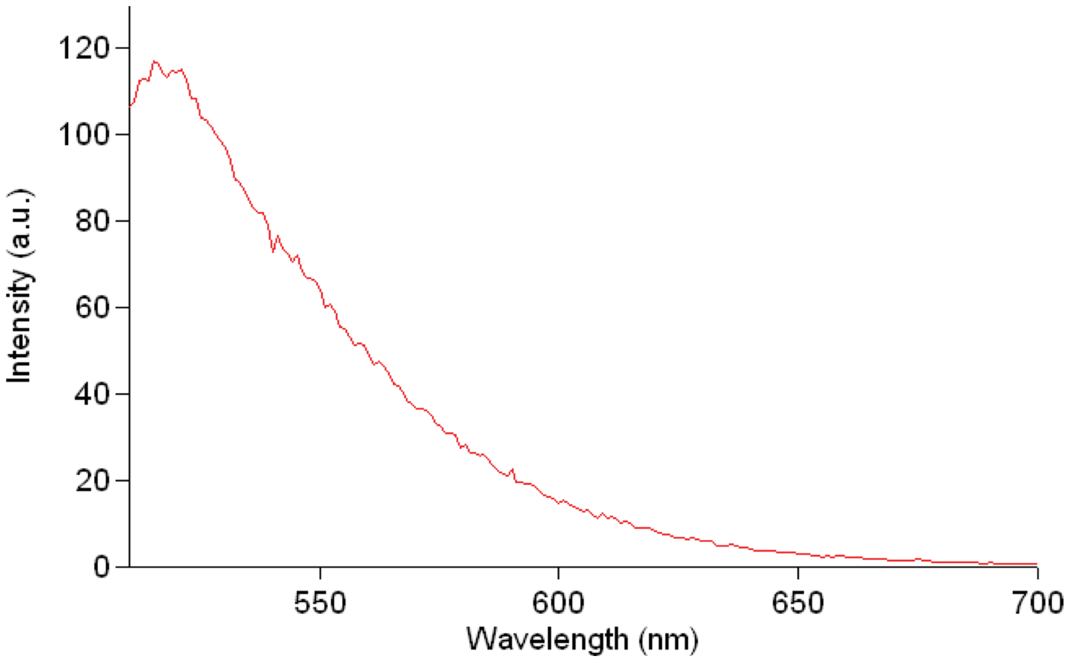

Supplementary Figure 5: The fluorescence spectrum of the OLICARB1:1 nanocapsules loaded with 5-carboxyfluorescein.
